# A promising clinical technique to rescue women’s ovarian aging: engineered stem cell-based therapy with the help of AI

**DOI:** 10.1093/procel/pwae047

**Published:** 2024-09-03

**Authors:** Yibo Wang, Huiyu Xu, Lin Fu, Yang Yu, Jie Qiao

**Affiliations:** State Key Laboratory of Female Fertility Promotion, Center for Reproductive Medicine, Department of Obstetrics and Gynecology, Peking University Third Hospital, Beijing 100191, China; National Clinical Research Center for Obstetrics and Gynecology, Peking University Third Hospital, Beijing 100191, China; Beijing Key Laboratory of Reproductive Endocrinology and Assisted Reproductive Technology, Peking University Third Hospital, Beijing 100191, China; Clinical stem cell research center, Peking University Third Hospital, Beijing 100191, China; State Key Laboratory of Female Fertility Promotion, Center for Reproductive Medicine, Department of Obstetrics and Gynecology, Peking University Third Hospital, Beijing 100191, China; National Clinical Research Center for Obstetrics and Gynecology, Peking University Third Hospital, Beijing 100191, China; Beijing Key Laboratory of Reproductive Endocrinology and Assisted Reproductive Technology, Peking University Third Hospital, Beijing 100191, China; Key Laboratory of Assisted Reproduction, Ministry of Education, Peking University, Beijing 100191, China; State Key Laboratory of Female Fertility Promotion, Center for Reproductive Medicine, Department of Obstetrics and Gynecology, Peking University Third Hospital, Beijing 100191, China; National Clinical Research Center for Obstetrics and Gynecology, Peking University Third Hospital, Beijing 100191, China; Beijing Key Laboratory of Reproductive Endocrinology and Assisted Reproductive Technology, Peking University Third Hospital, Beijing 100191, China; Clinical stem cell research center, Peking University Third Hospital, Beijing 100191, China; State Key Laboratory of Female Fertility Promotion, Center for Reproductive Medicine, Department of Obstetrics and Gynecology, Peking University Third Hospital, Beijing 100191, China; National Clinical Research Center for Obstetrics and Gynecology, Peking University Third Hospital, Beijing 100191, China; Beijing Key Laboratory of Reproductive Endocrinology and Assisted Reproductive Technology, Peking University Third Hospital, Beijing 100191, China; Clinical stem cell research center, Peking University Third Hospital, Beijing 100191, China; State Key Laboratory of Female Fertility Promotion, Center for Reproductive Medicine, Department of Obstetrics and Gynecology, Peking University Third Hospital, Beijing 100191, China; National Clinical Research Center for Obstetrics and Gynecology, Peking University Third Hospital, Beijing 100191, China; Beijing Key Laboratory of Reproductive Endocrinology and Assisted Reproductive Technology, Peking University Third Hospital, Beijing 100191, China; Key Laboratory of Assisted Reproduction, Ministry of Education, Peking University, Beijing 100191, China

As socioeconomic conditions improve and lifestyles change, the ratio of women giving birth at an advanced reproductive age (35–49 years) has increased dramatically (Qiao and Li, 2014). However, infertility caused by advanced age seriously affects women’s physical and mental health around the world. As the most important reproductive organ of women, the ovary is one of the earliest organs to appear aging. Ovarian aging in women is characterized by a decline in both the quality and quantity of oocytes, with significant individual differences in the timing and causes of these declines. The number of primordial follicles denotes the reserve of ovaries and determines the reproductive lifespan in women. In addition to that, the quality of oocytes is the most critical factor in determining female fertility (Park et al., 2021). Factors of ovarian aging are extremely complex. Lacking the precise information about the characterizations of ovarian aging and treating patients with non-personalized HRT, leading the traditional ART still remains limitations to be improved in a more precise way. Stem cells have the ability to unlimitedly proliferate and differentiate into diverse cell types while maintaining their characteristic of self-renewal, therefore, stem cell-based therapy offers hope for women with ovarian aging.

In recent years, advances in bioinformatics and AI have made it easier to identify increasing hallmarks of the ovarian aging including the timing of decline and the underlying molecular mechanisms, enabling the identification of potential therapeutic targets and the development of treatment strategies (Wang et al., 2020; Wu et al., 2024). The multi-omics datasets produced reveal the hallmarks of ovarian aging, precisely indicating the therapeutic targets. With the development of AI, many innovative AI tools have been applied to disease early diagnosis including reproductive medicine (Huang et al., 2023; Xu, 2023). OvaRePred is an outstanding AI tool used in reproductive medicine that estimates a woman’s current ovarian reserve and provides predictions for fertility milestones, including the age of diminished ovarian reserve (DOR) and the initiation of perimenopause, helping us to understand the characteristics of ovarian aging more precisely (Xu et al., 2023).

Meanwhile, the combination of AI tools with stem cell-based therapy also greatly facilitates the generation and application of stem cells. Recently, the human-induced pluripotent stem cells (iPSCs) and iPSC-derived cells are reported to have inherent advantages and exhibit better efficacy in the treatment of diverse diseases including Parkinson’s disease, Alzheimer’s disease, stroke, and other diseases, making these cells good candidates for cellular therapeutics and individualized medicine (Mukherjee et al., 2021; Qiao et al., 2020). However, the quality control and characterization of iPSCs and iPSC-derived cells with gene editing pose significant challenges in the precise selection of the best morphological clones. It is not feasible to avoid errors in large-scale cultures using a manual approach. With the development of AI algorithms, including machine learning (ML) and deep learning (DL), it is hopeful that new approaches can be developed to overcome these limitations and ensure the safety and efficacy of iPSCs and iPSC-derived cells (Mukherjee et al., 2021).

The combination of AI, gene editing, and stem cell-based therapy promises to make precise cell therapy a promising approach for rescuing female reproductive health. Although these cutting-edge technologies are expected to encounter new challenges in the process of application, taking advantage of these novel technologies and establishing a precise disease diagnosis and treatment strategy will guarantee the safety and efficacy of engineered stem cells, making stem cell-based therapy a promising clinical technique for addressing ovarian aging. In this perspective, we will introduce a clinical treatment strategy for rescuing ovarian aging using engineered stem cells.

## Identifying the hallmarks of ovarian aging

Identifying the hallmarks of ovarian aging, including ovarian reserve and the underlying molecular mechanisms, more precisely is therefore key to rescuing ovarian aging more effectively. In recent years, advances have made it possible to address this key issue using advanced bioinformatics and AI tools, which guide us in developing effective therapeutic strategies.

Primordial follicles remain in a dormant state for decades in women. These dormant follicles are the ovarian reserve that contributes eggs for the whole reproductive life span of females. The decline of the quantity and quality of follicles, known as ovarian aging, leads to a decrease in the ovarian reserve, an important indicator of women’s fertility (Camaioni et al., 2022; Park et al., 2021). The ovarian reserve varies significantly in different women even at the same age due to the individual differences, making it important to develop a method for predicting the ovarian reserve of women in a precise and personal way. Recently, we developed an innovative AI tool named OvaRePred in the field of reproductive health, which accurately predicts women’s current ovarian reserve with several parameters including serum Anti-Müllerian hormone (AMH), follicle-stimulating hormone (FSH), and age. Furthermore, this inspiring AI tool provides an assessment of key milestones in female fertility including the age of DOR and the initiation of perimenopause (Xu et al., 2023). With the easily obtained parameters, women can know more about their ovarian reserve and make a fertility plan with OvaRePred. For women with decreased ovarian reserve hoping to have a baby, clinical doctors can determine the time of treatment and appropriate interventions such as stem cell therapy based on the ovarian reserve obtained from OvaRePred and other valuable information. The predicted ovarian reserve provided by OvaRePred offers more precise guidelines about whether and when to treat patients with stem cells. While OvaRePred aids in the application of stem cell-based therapy to reproductive medicine, it is the underlying molecular mechanisms that guide us in generating functional stem cells and improving the efficacy of stem cell-based therapy for ovarian aging.

Therefore, uncovering the molecular mechanisms of ovarian aging and female age-related fertility decline is critical for precision medicine of stem cell-based therapy. In mammals, ovarian aging appears molecular and cellular changes in the quantity and quality of oocytes (Wu et al., 2024). The quantity and maturity of ovulated oocytes, as indicated by the release of the first polar body (PB1), were significantly reduced in older mice compared to the younger ones. Conversely, the rate of fragmentation was notably higher in the aged mice. It is the quality of oocytes that determines the female fertility. Many researchers revealed that the decline in oocyte quality due to age is accompanied by a significant decrease in the level of antioxidant genes, oxidative phosphorylation, and impairing metabolic homeostasis (Wang et al., 2020; Zhang et al., 2023). As previously reported by researchers, these factors are crucial for the development and maturation of oocytes. To improve the fertility of older women, recent studies have shown that supplementation with nicotinamide mononucleotide, spermidine, melatonin, and other drugs counteract decaying fertility of aged female mice by improving oocyte quality (Miao et al., 2020; Reiter et al., 2023; Zhang et al., 2023). These cutting-edge studies offer insights for clinical practice, suggesting that supplementation with drugs could enhance the reproductive success of older women, whether through natural pregnancy or assisted reproductive technology.

Besides the oocyte, other cell types as well as environmental factors contributing to ovarian aging, suggesting that ovarian decay is a complex and multifaceted process. With the single-cell transcriptomic landscape of ovaries from young and aged non-human primates, we identify seven ovarian cell types with distinct gene-expression signatures including oocyte and six types of ovarian somatic cells, uncovering cell-type-specific inactivation of antioxidant genes in aged monkey and human ovaries (Wang et al., 2020). In addition to this, recent study has mapped the spatiotemporal single-cell transcriptomic landscape of human ovarian aging and identified FOXP1 as deregulated with aging and modulating ovarian reserve. It is valuable to get the atlas data of human ovaries, from young and older women (Wu et al., 2024). These transcriptomic data still remain to be further analyzed for investigating more hallmarks of human ovarian aging, providing potential targets to rescue and delay ovarian aging. These studies provide comprehensive understanding about mammalian ovarian aging at cellular and molecular levels, which reveals new diagnostic biomarkers and potential therapeutic targets for age-related human ovarian disorders. Another study reveals the decline of ovarian mesenchymal progenitor (oMPs) number and differentiated stromal mesenchymal cells (dsMCs) aging at the molecular level leading to ovarian aging by analyzing the single-cell transcriptomic data of ovarian mesenchymal cells from young and aged mice. By transplanting the ovarian mesenchymal cells from young mice into ovaries of aged mice has been shown to remarkably rescue ovarian aging (Jia et al., 2023). What’s more, mitochondria and oxidative stress, changes in extracellular matrix molecules also play important roles in regulating the initiation of ovarian aging (Camaioni et al., 2022; Park et al., 2021). These interesting works indicate us that identifying the hallmarks of ovarian aging in other cellular components and rescuing the ovarian microenvironment with function-enhancing cells that target regressing non-follicular cells is a promising approach to improve ovarian function and slow down aging.

The human-induced pluripotent stem cells (iPSCs) have the inherent advantages to be modified and generate various types of function-enhancing cells, leading them to be a good tool for rescuing ovarian aging. With the great improvements in the genome editing technique CRISPR/Cas9, the gene-editing tools have been popular technologies for the investigation of the molecular and cellular mechanisms underlying various diseases (Sharma et al., 2021). Researchers have also found it effective to correct genomic mutations and modify specific functional genes. The integration of therapeutic targets with the gene-editing tool CRISPR/Cas9 guides us in understanding how to create cells that enhance specific functions to address ovarian dysfunction, ensuring both efficacy and safety. These cells are known as ovarian rescue cells (OvaResCells).

## Generation of ovarian rescue cells

Previous clinical studies have reminded us that mesenchymal stem cells (MSCs) therapy improves pregnancy outcomes in patients with premature ovarian failure (a type of pathological ovarian aging) slightly but significantly (Ding et al., 2018; Yan et al., 2020). These pioneering studies shed light on stem cell-based therapy in rescuing the ovarian aging of those hard to be cured by traditional ART. Due to limitations of MSCs, such as senescence, unclear targets, and heterogeneity among different sources, current stem cell therapies aimed at rescuing ovarian aging are still far from clinical application.

Recently, some researchers have proposed that human induced pluripotent stem cells (iPSCs), which have inherent advantages that might overcome the limitations of MSCs for cell therapy. The iPSCs are induced from various types of human somatic cells such as fibroblast, blood, and renal epithelial cells from the urine which can be easily obtained. After purifying, expanding, and further differentiation, the iPSCs derivatives still remain higher cell viability of proliferation, which can be further used in treatment with high efficiency.

**Figure 1.**
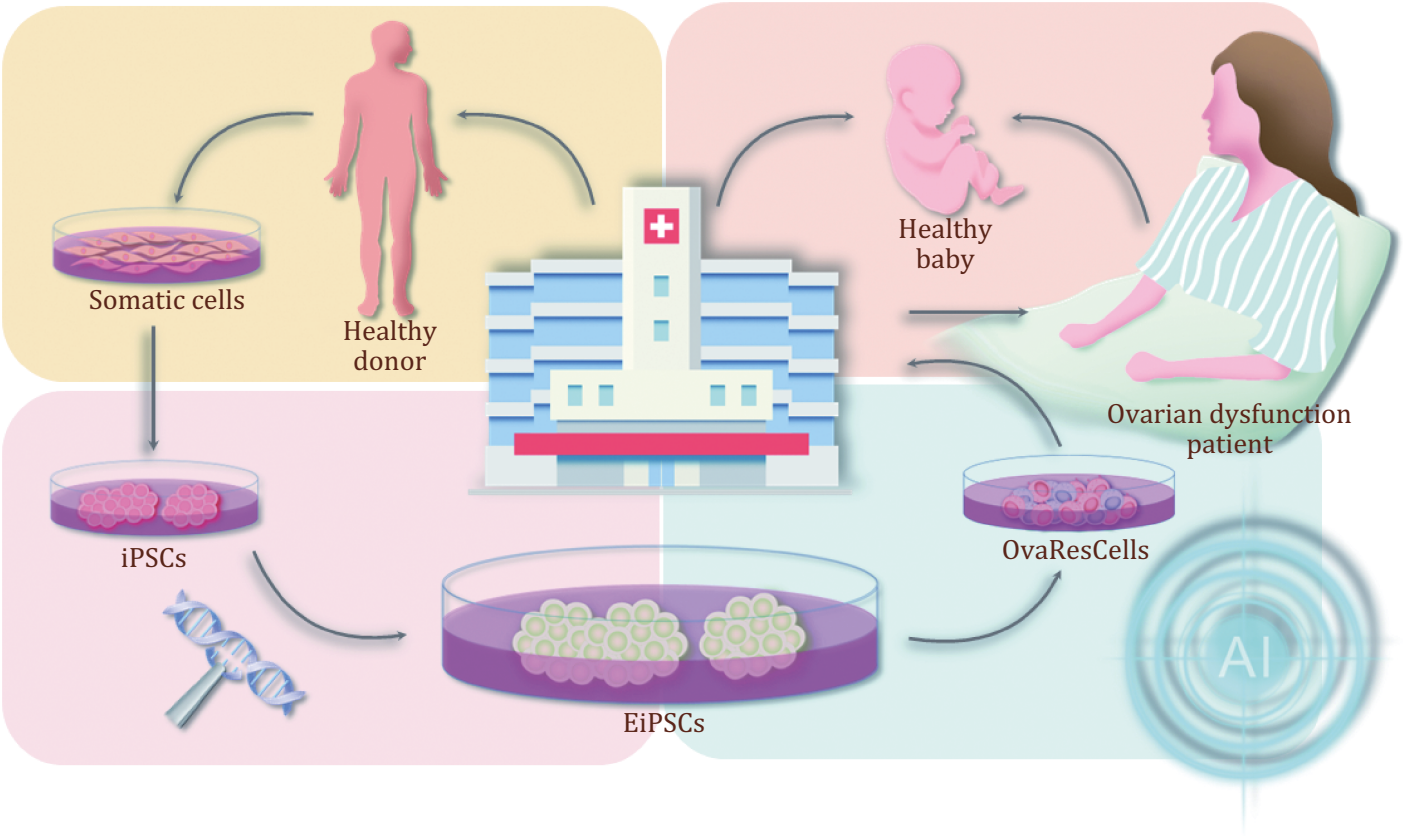
**The generation and application of OvaResCells in rescuing ovarian dysfunction.** OvaResCells is the abbreviation for “ovarian rescue cells”. The generation of OvaResCells is under the guidance of doctors and researchers using AI and gene-editing tools. The somatic cells are donated by healthy volunteers who come to the hospital for medical checkups. Reprogramming the somatic cells with defined factors and then strictly selecting the resulting human induced pluripotent stem cells (iPSCs). The expression of specific target genes is enhanced using a gene-editing tool, therefore generating and selecting the function-enhancing iPSCs (EiPSCs), which are further differentiated into function-enhancing cells, the ovarian rescue cells (OvaResCells), with AI tools. After injection with OvaResCells, women’s ovarian functions are expected to be significantly improved. The application of OvaResCells holds promise for rescuing ovarian dysfunctions and ensuring women’s reproductive health with safety and efficacy.

For generating the OvaResCells, it is important to select seed cells, the cell sources of iPSCs. The somatic cells maintain their genomic characteristics, including genomic instability such as single nucleotide variation (SNV) and single nucleotide polymorphism (SNP). Selecting seed cells with superior quality ensures that OvaResCells function more safely and effectively. Based on a large dataset including multi-omics, basic clinical examinations, and family history of illness collected from hospitals, it is relatively easier for researchers to select seed cells with high quality. Utilizing kits like Sendai Virus Reprogramming Kit to reprogram fibroblasts, blood cells, and other somatic cells into induced pluripotent stem cells (iPSCs) (Ban et al., 2011; Qiao et al., 2020; Yu et al., 2009). It utilizes Sendai virus particles to deliver key reprogramming factors including Oct3/4, Sox2, L-Myc, and Klf4, characterized by a high success rate and rapid RNA vector clearance (Okita et al., 2011). The Sendai virus is a non-integrating retrovirus, which means it can introduce genetic material into cells without integrating into the host genome, making it a preferred choice for certain types of cell-reprogramming applications due to its safety profile (Ban et al., 2011; Okita et al., 2008). With the strict screening criteria including clonal morphology and genomic stability, the iPSC clones with high quality are selected and expanded in another culture system. Finding the potential therapeutic target gene of ovarian aging, modifying the gene of iPSCs with gene-editing tool CRISPR/Cas9 is a fundamental way to realize the specific functional enhancement of iPSCs (EiPSCs). The EiPSCs are then picked out and treated with specific factors for further inducing different cell types like OvaResCells, which are function-enhancing cells that are strictly produced according to the guidelines of Good Manufacturing Practice of Medical Products (GMP). Choosing the appropriate time of clinical treatment with the help of OvaRePred and transplanting the OvaResCells into patients with ovarian aging, therefore, the OvaResCells and their byproducts perform the specific functions aiming to specific ovarian cells and improve the quality and quantity of follicles *in vivo*. This strictly controlled pipeline of cell production makes it promising for women to give birth with this OvaResCell-based therapy (Fig. 1).

However, this approach of OvaResCell-based therapy faced many challenges related to the enormous amount of data analysis, rising quantity and complexity, standardization methods, manual errors, and extremely difficult data handling, which poses a significant challenge to guarantee the scalability, reproducibility, and consistency of iPSCs and iPSC-derived cells (Mukherjee et al., 2021). By taking advantage of developed AI algorithms in recent years, many researchers tried to install an automatic approach for accurate segmentation and colony quality control to solve these challenges. The machine learning (ML) and deep learning (DL) are two widely adopted AI algorithms in the field of medical health. In the case of selecting iPSC clones with high-quality, convolutional neural network is developed and trained to distinguish among differentiated and undifferentiated cells with an accuracy of 99%. In addition, the supervised ML algorithms commonly used in the medical field include artificial neural network (ANN), support vector machine (SVM), random forest (RF), k-Nearest Neighbors, decision tree (DT), adaptive boosting (Adaboost), among others, showing the ability to assess automated texture descriptors of segmented colony regions of iPSCs and to check their potential. Among these tools, SVM, RF, and Adaboost classifiers have been concluded to exhibit superior classification ability over ML and DT.

Combining those advanced AI tools with stem cell-based therapy, it is possible to overcome the limitations of the conventional approach of generating iPSCs and iPSCs derivates, enabling accuracy segmentation of iPSCs clones with high-quality. Nowadays, CRISPR/Cas9 is one of the most widely used gene-editing tools in clinical treatment (Sharma et al., 2021), it is a trend to establish a new approach to generate function-enhancing iPSCs with CRISPR/Cas9 automatically. Considering the multiple challenges in estimating the safety and efficacy of gene editing in iPSCs and accurately selecting differentiated cells from iPSCs, however, requires a substantial amount of data support with basic study and manual operation in generating clinic-level function-enhancing cells. Therefore, new AI algorithms of ML and DL that are urgently needed for the automated generation of OvaResCells with gene-editing tool (Fig. 1). In summary, it is the development of AI tools that guarantees the safety and efficacy of generated OvaResCells with gene-editing tool CRISPR/Cas9 and make OvaResCell-based therapy possible for the application into precision reproductive medicine in a few years.

## Delivery of OvaResCells

The delivery of OvaResCells refers to the method by which these cells are transported and administered to the specific location within the ovaries. Effective delivery is essential for the therapeutic success of OvaResCells, ensuring they reach the intended site and can exert their potential regenerative effects on the ovarian tissue. Organ site injection and intravenous injection are two prevalent methods for administering stem cells, each presenting unique benefits and drawbacks. Organ site injection offers the advantages of targeted action and focused efficacy, particularly for diseases localized to specific organs. However, it requires a more complex procedure and carries the risk of potential side effects if not performed accurately. On the other hand, intravenous injection is simpler to administer and has a broad range of applications, especially for systemic diseases or conditions affecting multiple organs. Yet, it may be hindered by issues with homing efficiency and the pulmonary first-pass effect, which can reduce the overall therapeutic impact (Fischer et al., 2009; Yu et al., 2023).

In clinical decision-making, the choice of injection route should be tailored to the disease type, treatment goals, patient status, and anticipated outcomes. In the case of delivering of OvaResCells to rescue female’s fertility, organ site injection might be preferred for localized injuries or organ-specific diseases due to its precision, whereas intravenous injection could be more appropriate for systemic or multi-organ conditions due to its ease of administration. Recent clinical studies have demonstrated the effectiveness of organ site injection as a delivery method by administering MSCs through direct ovarian injection, achieving the delivery of stem cells to the target organ and rescuing ovarian function (Ding et al., 2018; Pei et al., 2024; Yan et al., 2020). In addition to the way of delivery, to enhance treatment efficacy, optimizing stem cell quality and homing capabilities is crucial, as demonstrated by clinical studies that highlight the importance of these factors in improving patient outcomes.

Furthermore, to evaluate the effectiveness of OvaResCell-based therapy for ovarian aging, in addition to the final pregnancy outcomes, it is necessary to design stringent clinical protocols to assess the changes in various physiological indicators of female ovaries before and after OvaResCells treatment. This includes the levels of serum AMH, FSH, and estradiol (E2), the volume of the ovaries, ovarian stromal blood flow, and the count of antral follicles, among other routine clinical indicators.

## Conclusion

The progress made in artificial intelligence and bioinformatics has promoted our cognition of ovarian aging in a more precise way, leading us to comprehensively understand the hallmarks of ovarian aging and reveal the underlying molecular mechanisms and potential therapeutic targets, giving us guidelines about whether and when to initiate patients with OvaResCell-based therapy. Effectively integrating tools like artificial intelligence and gene editing is the essential path to realizing precision medicine in the cellular therapy of OvaResCells. In summary, artificial intelligence is deeply integrated throughout the entire process of OvaResCells, from production to application. Utilizing bioinformatics and clinical big data, combined with the machine learning and deep learning algorithms of artificial intelligence, we can identify precise targets for treating female ovarian aging and the best seed cells. Designing bioinformatics analysis combined with machine training models allows for the morphological and gene expression detection of reprogrammed iPSC cells and gene-edited EiPSCs. This process enables the selection of the best EiPSC cells and iPSC-derived cells from both morphological and molecular perspectives. Furthermore, the OvaRePred artificial intelligence model can achieve personalized differential diagnosis. By integrating clinical testing data, physicians can determine whether patients need stem cell therapy and the optimal timing for treatment.

Stem cell therapy combined with gene editing, as a cutting-edge medical technology, has demonstrated tremendous potential and hope in preclinical research. However, the clinical translation of stem cell therapy combined with gene editing faces a multitude of challenges and gaps, ranging from technological complexity and safety concerns to regulatory hurdles, ethical dilemmas, cost implications, efficacy validation, public acceptance, and the need for robust data management and supply chain logistics. Overcoming these challenges requires a comprehensive application of multi-faceted strategies, including enhancing foundational research, technological innovation, interdisciplinary collaboration, well-designed clinical trials, rational regulation and policy formulation, ethical review, cost control, patient education, data sharing, long-term monitoring, international cooperation, risk management, and supply chain optimization. And, utilizing AI tools is an effective way to accelerate the overcoming of challenges in the medical field and to bridge the gap between knowledge and technology.

In this perspective, we introduce the concept of a novel precision stem cell therapy for ovarian aging, known as OvaResCells. This perspective systematically elaborates on the process from identifying target genes for iPSCs, generating OvaResCells, to delivering them to the ovaries. We explore OvaResCells as a potential therapeutic method to save women’s fertility health, combining technologies such as stem cells, gene editing, and artificial intelligence. By enhancing preclinical evidence of effectiveness and elucidating specific molecular mechanisms, and by addressing the multiple challenges encountered during the application process, it is promising to establish OvaResCells as guardians of women’s reproductive health.
